# Role of ferredoxin 1 (FDX1) in cancer and its therapeutic potential

**DOI:** 10.1016/j.cpt.2025.11.002

**Published:** 2025-11-07

**Authors:** Fen He, Hongyan Zhao, Ruixin Gao, Maoyou Lu, Siqi Wang, Yu Chen, Lijun Peng, Jiliang Xia

**Affiliations:** a Hunan Province Key Laboratory of Tumor Cellular & Molecular Pathology, Cancer Research Institute, Hengyang Medical School, University of South China, Hengyang, Hunan 421001, China; b Department of Spine Surgery, The First Affiliated Hospital, Hengyang Medical School, University of South China, Hengyang, Hunan 421001, China

**Keywords:** *FDX1*, Biomarker, Cancer progression, Cuproptosis, Cancer therapy

## Abstract

Ferredoxin 1 (FDX1) is a small iron-sulfur (Fe–S) cluster protein localized to the mitochondria. It functions as an electron carrier in diverse metabolic pathways and is critically involved in the regulation of protein lipoylation Accumulating evidence indicates that *FDX1* expression is frequently dysregulated across various cancer types. Its expression is significantly associated with cancer progression, prognosis, and tumor immune responses, suggesting its potential as a biomarker for cancer diagnosis, prognostic assessment, and prediction of immunotherapy response. Transcription factors, epigenetic modifications, and non-coding RNAs (ncRNAs) contribute to the aberrant expression of *FDX1* in cancer cells. Mechanistic studies reveal that FDX1 protein influences cancer progression by modulating oncogenic signaling pathways, metabolic reprogramming, and tumor immunity: Notably, FDX1 serves as a central mediator of cuproptosis — a copper-dependent form of programmed cell death — highlighting its potential tumor-suppressive function. Therefore, FDX1 may exert dual roles in cancer by either promoting or inhibiting disease progression. Recently, several agents targeting FDX1 have exhibited promising therapeutic efficacy both *in vitro* and *in vivo* across diverse cancer models. In this review, we summarize the regulatory mechanisms governing of *FDX1* expression and its functional roles in cancer progression, and further highlight its potential as a therapeutic target for cancer.

## Highlights

• Ferredoxin 1 (*FDX1*) expression is dysregulated in various cancer types, either upregulation or downregulation.
• FDX1 plays dual roles in different cancer types, exhibiting both tumor-promoting and tumor-suppressive functions
• FDX1 is a key mediator of cuproptosis; therefore, elevating its expression or activity sensitizes cancer to cuproptosis.
• *FDX1* expression correlates with tumor immunity, suggesting it is a potential biomarker and therapeutic target for immunotherapy.

## Graphical abstract

**Figure:**
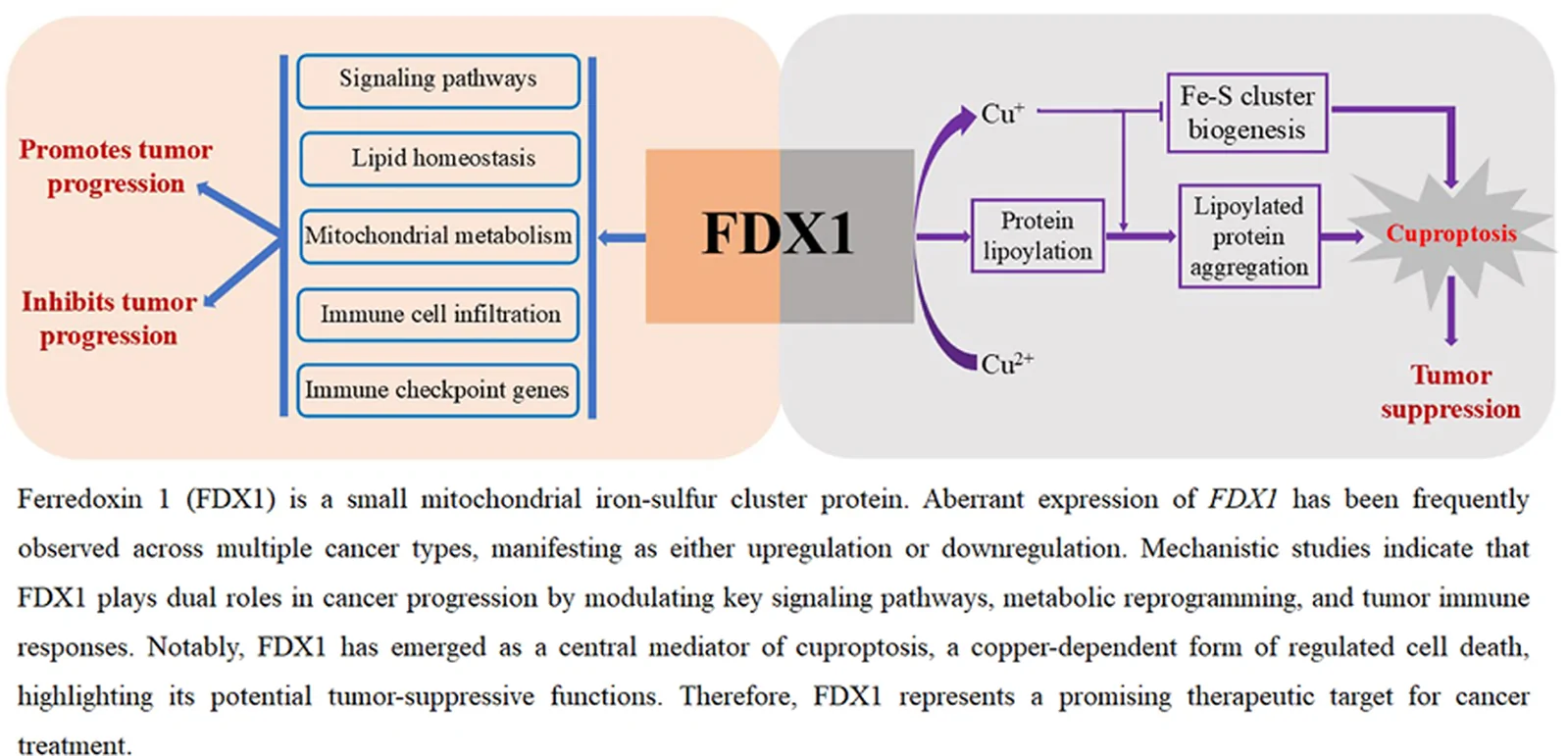

## Introduction

Ferredoxins (FDXs) are evolutionarily conserved iron-sulfur (Fe–S) cluster-containing proteins localized in the mitochondria, where they serve as electron transfer mediators in various metabolic pathways. In mammals, the two ferredoxin isoforms, FDX1 and FDX2, share 43% sequence identity and possess highly similar structural characteristics.^1^, ^2^, ^3^ Evidence increasingly supports a specific role for FDX2 in Fe–S cluster biogenesis.^1^ By contrast, FDX1 participates in a diverse range of biological functions, including protein lipoylation, Fe–S cluster biosynthesis, steroid and bile acid synthesis, heme α/α3 formation, vitamin A and D metabolism, and copper ion reduction.^1^

*FDX1* expression is dysregulated in various cancers, such as gastric cancer,^4^ hepatocellular carcinoma (HCC),^5^ breast cancer,^6^ colorectal cancer,^7^ clear cell renal cell carcinoma (ccRCC),^8^ and glioma.^9^ This aberrant expression is significantly associated with cancer progression, patient prognosis, and tumor immune responses, suggesting that *FDX1* may serve as a promising biomarker for cancer diagnosis, prognostic evaluation, and immunotherapy response prediction. Multiple factors contribute to the altered *FDX1* expression in these malignancies, such as regulation by transcription factors,^7^^,^^10^, ^11^, ^12^, ^13^ epigenetic modifications,^7^^,^^14^, ^15^, ^16^, ^17^, ^18^ and non-coding RNAs (ncRNAs).^19^, ^20^, ^21^, ^22^, ^23^, ^24^ The FDX1 protein plays a critical role in key cancer-related processes, including cell proliferation, drug resistance, radioresistance, invasion, and metastasis across multiple tumor types.^8^^,^^15^^,^^25^, ^26^, ^27^ Mechanistically, FDX1 modulates cancer progression by regulating oncogenic signaling pathways,^5^^,^^28^, ^29^, ^30^ metabolic reprogramming,^31^, ^32^, ^33^ and tumor immunity.^34^^,^^35^ Under conditions of copper overload, FDX1 reduces Cu^2+^ to the toxic Cu^+^ form, promoting the aggregation of lipoylated proteins and inhibiting Fe–S cluster biogenesis, which subsequently induces cuproptosis—a copper-dependent form of programmed cell death.^36^ Therefore, FDX1 exerts a dual role in cancer, acting either as a promoter or suppressor of cancer progression depending on the cancer type. Given its central involvement in oncogenesis and cuproptosis, several anticancer strategies targeting FDX1 directly or indirectly have been developed, showing substantial therapeutic potential in various malignancies.^11^^,^^37^, ^38^, ^39^, ^40^ This review aims to summarize the current understanding of *FDX1* dysregulation and its regulatory mechanisms in cancer, outlines the molecular pathways through which FDX1 protein influences cancer progression, and discusses the therapeutic potential of targeting FDX1. Furthermore, it highlights future research directions for *FDX1* studies and the development of novel anticancer therapeutic strategies.

## Physiological functions of ferredoxin 1 (FDX1) in normal cells

**Figure 1:**
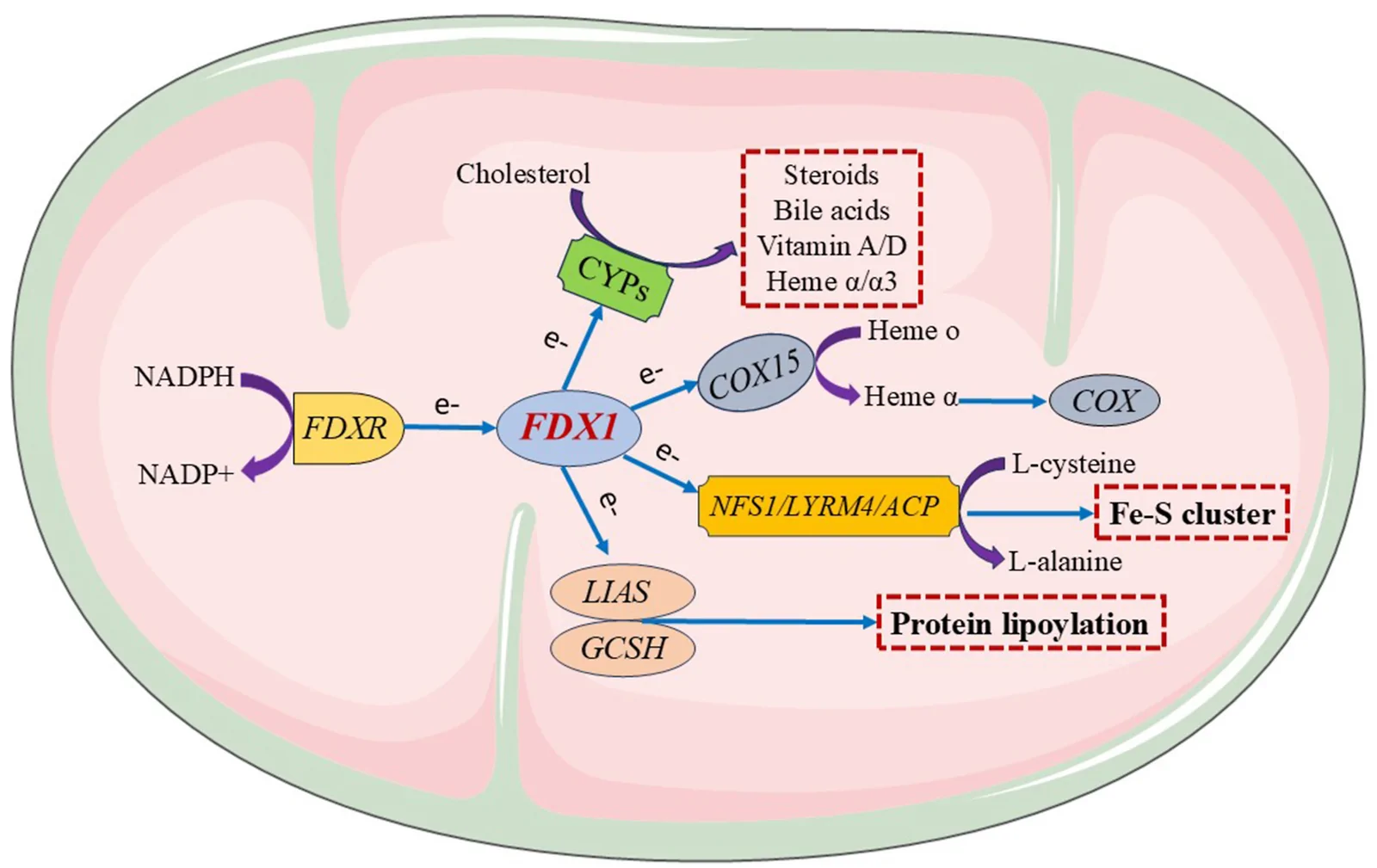
The physiological functions of FDX1. FDX1 receives electrons from NADPH via FDXR and subsequently participates in various cellular processes by donating these electrons to downstream mitochondrial acceptors. It donates electrons to mitochondrial cytochrome P450 enzymes (CYPs), thereby initiating the biosynthesis of steroids, bile acids, vitamin A and D, and heme a/a3. Furthermore, FDX1 modulates COX activity by delivering electrons to COX15, the heme α synthase essential for COX assembly and function. In addition, FDX1 interacts with the NFS1–LYRM4–ACP complex to facilitate the conversion of l-cysteine to l-alanine and support Fe–S cluster biogenesis via electron transfer. FDX1 also functions as an upstream regulator of protein lipoylation by binding to LIAS and enhancing its functional interaction with the lipoyl carrier protein GCSH. ACP: Acyl carrier protein; COX: Cytochrome c oxidase; COX15: Cytochrome *c -* oxidase assembly homolog 15; FDX1: Ferredoxin 1; FDXR: Ferredoxin reductase; GCSH: Glycine cleavage system H-protein; LIAS: Lipoyl synthase; LYRM4: LYR motif containing 4; NADPH: Nicotinamide adenine dinucleotide phosphate; NFS1: Nitrogen fixation 1.

The human *FDX1* is located on chromosome 11q22.3 and consists of two exons encoding a 184-amino-acid protein with a molecular weight of 19.4 kDa. The FDX1 protein participates in multiple biological processes in mammalian cells [Figure 1]. Its canonical function lies in steroid biosynthesis, where FDX1 receives electrons from NADPH via ferredoxin reductase (FDXR), an NADPH-dependent flavoprotein, and transfers these electrons to mitochondrial cytochrome P450 enzymes (CYPs), thereby initiating steroidogenesis.^41^^,^^42^ The mitochondrial FDX1-CYP system not only drives steroid biosynthesis but also regulates the production of bile acids, heme α/α3, and vitamins A/D^42^. FDX1 depletion reduces both the expression and activity of cyclooxygenase (COX), a heme α/α3-dependent enzyme, in human cells, underscoring its regulatory role in COX function.^42^^,^^43^ Mechanistic studies indicate that FDX1 regulates COX by delivering electrons to cytochrome *c* oxidase assembly homolog 15 (COX15), the heme α synthase.^42^ FDX1 also interacts with the cysteine desulfurase complex involved in Fe–S cluster biogenesis, which consists of cysteine desulfurase, LYR motif-containing 4, and acyl carrier protein.^2^ Within this complex, FDX1 donates electrons to facilitate the conversion of l-cysteine to l-alanine, leading to Fe–S cluster generation.^2^ As an upstream regulator of cellular protein lipoylation, a mitochondrial lipid-dependent post-translational modification, FDX1 interacts with lipoyl synthase (LIAS) and facilitates its association with the lipoyl carrier protein glycine cleavage system H (GCSH).^33^ Lipoylation is critical for the enzymatic activity of four mitochondrial multienzyme complexes: pyruvate dehydrogenase, α-ketoglutarate dehydrogenase, branched-chain α-ketoacid dehydrogenase, and the glycine decarboxylase complex.^33^ Thus, FDX1 plays a central role in the tricarboxylic acid (TCA) cycle, branched-chain amino acid (BCAA) catabolism, and the glycine cleavage pathway.^33^ FDX1-mediated lipoylation not only supports mitochondrial metabolism but also generates substrates required for cuproptosis. Under conditions of copper overload, FDX1 reduces Cu^2+^ to Cu^+^, which promotes the aggregation of lipoylated proteins and inhibits Fe–S cluster biogenesis, ultimately inducing cuproptosis.^36^ Consequently, FDX1 acts as a key mediator of cuproptosis. Overall, FDX1 functions as an electron transfer protein and reductase that participates in diverse metabolic pathways and plays a role in cuproptosis.

## Aberrant expression of ferredoxin 1 (*FDX1*) and its regulatory mechanisms in human cancer

**Figure 2:**
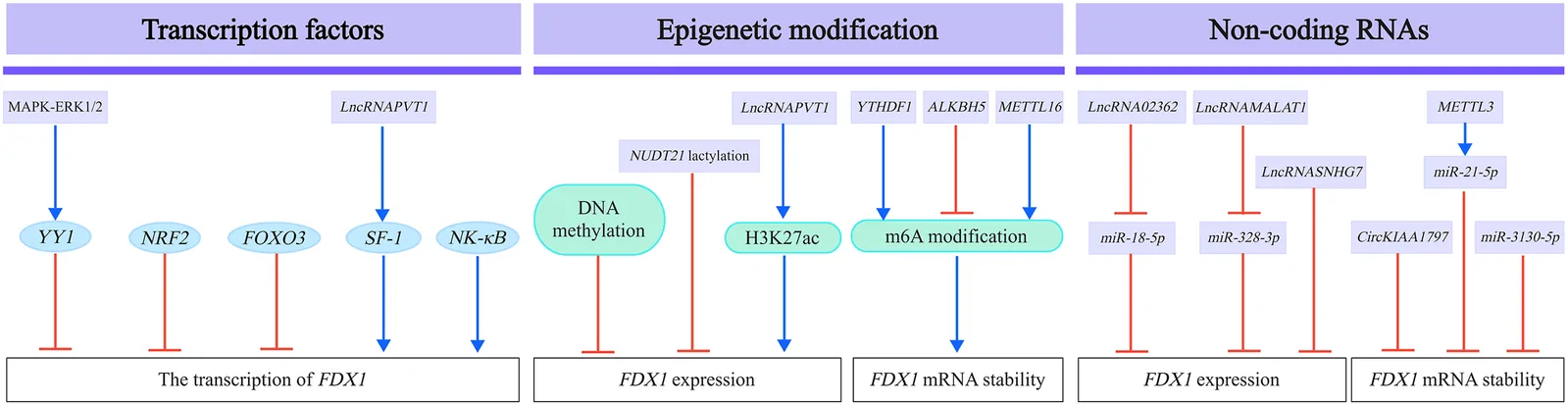
The regulatory mechanisms underlying aberrant *FDX1* expression in cancer. *FDX1* expression is regulated by multiple transcription factors: *YY1*, *NRF2*, and *FOXO3* act as transcriptional repressors, whereas *SF-1* and *NF-κB* function as activators. *FDX1* expression levels are inversely correlated with the methylation status of its promoter region across various cancer types. Lactylated NUDT21 suppresses FDX1 expression by promoting 3′ UTR elongation. The lncRNA PVT1 enhances *FDX1* transcription through direct binding to its promoter region and facilitates the enrichment of H3K27ac, a histone modification associated with active enhancers. *YTHDF1* and *METTL16* positively regulate *FDX1* expression by promoting m6A modification of its mRNA, while ALKBH5 negatively regulates *FDX1* expression by reducing m6A levels. In addition, *FDX1* expression is modulated by non-coding RNAs, including lncRNAs, circRNAs, and miRNAs. *ALKBH5*: AlkB homolog 5, RNA demethylase; circRNAs: Circular RNAs; *FDX1*: Ferredoxin 1; *FOXO3*: Forkhead box O-3; lncRNAs: Long non-coding RNAs; *METTL16*: Methyltransferase-like protein 16; miRNAs: microRNAs; *NF-κB*: Nuclear factor-κB; *NRF2*: Nuclear factor erythroid 2-related factor 2; *NUDT21*: Nudix hydrolase 21; *SF-1*: Steroidogenic factor-1; YTHDF1: YTH domain-containing family protein 1; *YY1*: Yin Yang 1; 3′-UTR: 3′-untranslated region.

Given the critical role of the *FDX1* in multiple cellular processes, normal cells maintain tight regulation of its expression. However, in numerous cancer types, *FDX1* expression is frequently dysregulated at both the mRNA and protein levels. This dysregulation is significantly associated with either poor or favorable prognosis, underscoring the potential of FDX1 as a prognostic biomarker [Table 1]. Several regulatory mechanisms contribute to aberrant *FDX1* expression across different cancers, including transcription factors, epigenetic modifications, and ncRNAs [Figure 2].

**Table 1:** Overview of *FDX1* expression in cancer tissues and cell lines.

*FDX1* expression	Cancer type	Sample type	Detection method	Biological function	References
Upregulation	Ovarian cancer	Tumor tissues, cell lines	TCGA, qRT-PCR, and IHC	Promotes progression and drug resistance	25,28
	Multiple myeloma	Tumor tissues, cell lines	GEO and qRT-PCR	Exhibits strong diagnostic value and promotes disease progression by modulating immune-related pathways	110
	Stomach adenocarcinoma	Tumor tissues	GEO, TCGA, and IHC	Acts as a diagnostic marker and is positively associated with favorable outcomes	16,111
	Glioma	Tumor tissues	TCGA, WB, and IHC	Positively associated with poor prognosis, immune cell infiltration, and immune checkpoint genes and mediates cuproptosis	34,45,112
	Uterine corpus endometrial carcinoma	Tumor tissues	TCGA	Positively associated with Poor prognosis and immune-related genes	15
	Osteosarcoma	Tumor tissues and cell lines	GEO and qRT-PCR	Positively associated with poor outcome and immune cell infiltration and promotes migration	113
Downregulation	Hepatocellular carcinoma	Tumor tissues	TCGA, GEO, IHC, qRT-PCR, and WB	Positively associated with favorable outcomes, inhibits tumor growth and metastasis, and mediates cuproptosis	5,44
	Papillary thyroid carcinoma	Tumor tissues	TCGA, GEO, and IHC	Negatively associated with tumor recurrence and positively associated with immune cell infiltration	114
	Colorectal cancer	Tumor tissues	TCGA and IHC	Positively ssociated with favorable clinical outcomes and immune cell infiltration, and mediates cuproptosis	7,96
	Clear cell renal cell carcinoma	Tumor tissues, cell lines	GEO, TCGA, IHC, and WB	Positively associated with favorable outcomes and immune infiltration, inhibits tumor growth and metastasis, and mediates cuproptosis	8,45,115
	Non-small cell lung cancer	Tumor tissues, plasma	TCGA, GEO, IHC, and ELISA	Acts as a diagnostic marker and is positively associated with favorable outcomes, inhibits cell proliferation, migration, and invasion	97,116,117
	Breast cancer	Tumor tissues	TCGA	Positively associated with favorable prognosis in pan-cancer analysis, induces malignant progression and immune suppression in triple-negative breast cancer	45,118
	Diffuse large B-cell lymphoma	Tumor tissues and cell lines	GEO and WB	Positively associated with favorable prognosis	29
	Kidney chromophobe	Tumor tissues	TCGA and GEO	Positively associated with favorable prognosis	45
	Cholangiocarcinoma	Tumor tissues and cell lines	TCGA, GEO, IHC, and qRT-PCR	Positively associated with favorable prognosis	119,120
	Head and neck squamous cell carcinoma	Tumor tissues	GEO, TCGA, and IHC	Negatively correlated with malignancy and T-cell exhaustion	45
	Kidney renal papillary cell carcinoma	Tumor tissues	GEO, TCGA, and IHC	Positively associated with favorable prognosis	45
	Adrenocortical carcinoma	Tumor tissues	GEO, TCGA, and GTEx	Act as an independent prognostic predictor and is associated with poor prognosis, immune infiltration and immune checkpoints	121
	Esophageal squamous cell carcinoma	Tumor tissues	GEO and TCGA	Associated with poor prognosis	122

ELISA: Enzyme-linked immunosorbent assay; *FDX1*: Ferredoxin 1; GEO: Gene Expression Omnibus; GTEx: Genotype-Tissue Expression Project; IHC: Immunohistochemistry; qRT-PCR: Quantitative reverse transcription-polymerase chain reaction; TCGA: The Cancer Genome Atlas; WB: Western blot.

### Transcription factors

Gene expression analyses from the Gene Expression Omnibus and The Cancer Genome Atlas databases show that *FDX1* mRNA levels vary widely across various cancer types, indicating transcriptional regulation of *FDX1* in cancer cells.^44^^,^^45^

Yin Yang 1 (*YY1*), a transcription factor belonging to the GLI-Kruppel family of zinc-finger proteins, plays a critical role in tumor initiation and progression.^46^ Wu et al. reported that SEC14-like lipid binding 3 induces cuproptosis by upregulating *FDX1* expression through inhibition of the mitogen-activated protein kinase–extracellular signal-regulated kinase 1/2-YY1 signaling axis, revealing *YY1* as a transcriptional repressor of *FDX1*.^12^ Nuclear factor erythroid 2-related factor 2 (*NRF2*), a transcription factor central to oxidative stress responses, also functions as a transcriptional inhibitor of *FDX1* in cervical cancer cells.^11^ Additionally, phosphorylated forkhead box O3 (*FOXO3*) translocates to the nucleus and suppresses *FDX1* transcription in ccRCC cells, establishing *FOXO3* as another transcriptional repressor of *FDX1*.^13^ The collective inhibitory roles of *YY1*, *NRF2*, and *FOXO3* suggest that combining a cuproptosis inducer with an inhibitors of these transcription factors may represent a promising strategy to enhance therapeutic efficacy. Conversely, *FDX1* is transcriptionally upregulated in multiple cancer types. Steroidogenic factor-1 (*SF-1*) binds to the *FDX1* promoter and enhances its transcriptional activity in colorectal cancer cells, a process facilitated by the long non-coding RNA (lncRNA) *PVT1*.^7^ Moreover, nuclear factor kappa B (*NF-κB*) acts as a positive transcriptional regulator of *FDX1* by binding to its promoter. Knockdown of *NF-κB* significantly suppresses tumor growth, while co-overexpression of *FDX1* partially reverses this effect in both *in vitro* and *in vivo* glioblastoma models.^10^ The transcriptional activation of *FDX1* by SF-1 and *NF-κB* highlights a potential therapeutic strategy for cancers characterized by the activation of these factors. Overall, *YY1*, *NRF2*, and *FOXO3* function as transcriptional repressors of *FDX1*, whereas *SF-1* and *NF-κB* serve as transcriptional activators of *FDX1* in cancer.

### Epigenetic modifications

Aberrant epigenetic alterations can cause inappropriate upregulation or silencing of gene expression, contributing to cancer initiation and progression.^47^ Increasing evidence indicates that epigenetic modifications regulate *FDX1* expression.

Across multiple cancer types, *FDX1* expression shows a negative correlation with DNA methylation levels, suggesting that methylation contributes to *FDX1* downregulation.^15^^,^^48^ By contrast, N6-methyladenosine (m6A) modification, the most prevalent RNA epigenetic modification, enhances *FDX1* expression. The YTH N6-methyladenosine RNA-binding protein F1 (*YTHDF1*) promotes MYC-induced *FDX1* expression in glioma cells by facilitating m6A modification of *FDX1* mRNA,^14^ thereby supporting glioma cell proliferation and invasion through the MYC–YTHDF1–FDX1 axis.^14^ Moreover, copper stress induces lactylation of methyltransferase-like protein 16 (*METTL16*) at lysine 229, which upregulates *FDX1* expression by enhancing the m6A modification of *FDX1* mRNA in gastric cancer.^16^ Sirtuin 2 suppresses *METTL16* lactylation; therefore, combining elesclomol, a copper ionophore, with AGK2, a sirtuin 2 inhibitor, enhances cuproptosis in gastric cancer.^16^ Histone acetylation also affects *FDX1* expression. lncRNA *PVT1* binds to the *FDX1* promoter, increases acetylation of histone H3 at lysine 27, and activates *FDX1* transcription.^7^ Furthermore, lactate dehydrogenase A-induced lactylation of Nudix hydrolase 21 (NUDT21) extends the *FDX1* mRNA 3′-untranslated region (3′-UTR) and represses its expression by interacting with cleavage and polyadenylation specificity factor 6 (*CPSF6*) in esophageal squamous cell carcinoma (ESCC).^17^ The combination of stiripentol, a lactate dehydrogenase A inhibitor, and elesclomol synergistically suppresses ESCC growth by targeting the NUDT21-CPSF6-FDX1 axis.^17^ Overall, epigenetic modifications play a key role in *FDX1* dysregulation in cancer. The combination of cuproptosis inducers and epigenetic modulators may offer a promising therapeutic strategy for cancer treatment.

### Non-coding RNAs (ncRNAs)

NcRNAs are single-stranded RNA molecules that play critical roles in regulating gene expression and contribute significantly to cancer progression.^49^ Major types of ncRNAs include microRNAs (miRNAs), lncRNAs (long non-coding RNAs), and circular RNAs (circRNAs). Increasing evidence shows that ncRNAs regulate *FDX1* gene expression in various cancer types.

The oncogenic miRNA *miR-21-5p* directly binds to the 3′-UTR of *FDX1* mRNA, promoting its degradation and thereby facilitating ccRCC progression.^50^ The *miR-21-5p*–*FDX1* axis is also present in non-small cell lung cancer.^23^ Moreover, *miR-3130-5p* suppresses *FDX1* expression by binding to its 3′-UTR in HCC cells.^24^ These findings indicate that miRNAs are key negative regulators of *FDX1* expression in various cancers. By contrast, lncRNAs upregulate *FDX1* expression by targeting miRNAs. Specifically, lncRNA *02362* enhances *FDX1* by reducing *miR-18-5p* levels.^19^ Activation of the lncRNA *02362*–*miR-18-5p*–*FDX1* axis inhibits cell proliferation and increases oxaliplatin sensitivity through cuproptosis in HCC cells.^19^ Additionally, the lncRNA metastasis-associated lung adenocarcinoma transcript 1 (*MALAT1*) increases *FDX1* expression by suppressing miR-328–3p in stomach adenocarcinoma.^20^ Conversely, knockdown of the lncRNA small nucleolar RNA host gene 7 (*SNHG7*) enhances *FDX1* expression, which in turn inhibits proliferation, invasion, and metastasis by inducing cuproptosis in colorectal cancer.^21^ Beyond miRNAs and lncRNAs, circRNAs also regulate *FDX1* expression. An 8-oxoguanine-modified circRNA derived from *KIAA1797* binds to *FDX1* mRNA, reducing its stability and suppressing cuproptosis, thereby promoting lung cancer progression.^22^ Collectively, these results identify FDX1 as a downstream target of ncRNAs in multiple cancer types. The ncRNA-FDX1 regulatory axis plays a critical role in cancer progression, making it a promising therapeutic target for cancer treatment.

## Role of ferredoxin 1 (FDX1) in cancer progression

**Figure 3:**
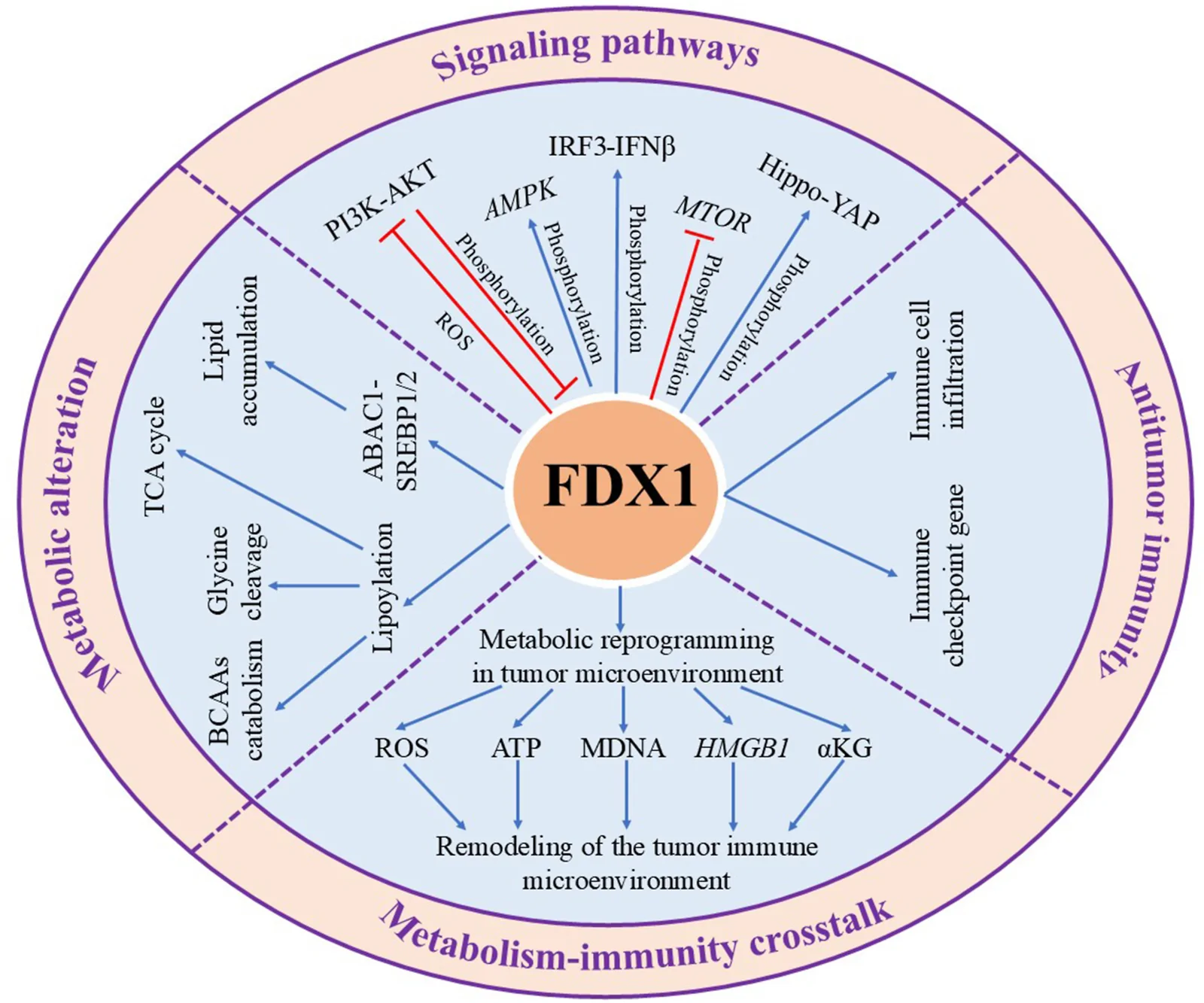
The underlying mechanisms by which FDX1 modulates cancer progression:signaling pathways, metabolic reprogramming, and tumor immunity. FDX1 plays a crucial role in cancer progression by modulating several key signaling pathways, including AMPK, mTOR, PI3K-AKT, IRF3-IFN-β, and Hippo-YAP. It is also essential for maintaining lipid homeostasis and regulating mitochondrial metabolism. Moreover, FDX1 expression is positively correlated with immune cell infiltration across various cancer types and is involved in the regulation of immune checkpoint genes, suggesting its potential involvement in antitumor immunity. Furthermore, FDX1 may contribute to remodeling the tumor immune microenvironment through the modulation of intratumoral metabolic processes. PI3K–AKT: Phosphatidylinositol 3'-kinase(PI3K)–Protein kinase B (AKT); AMPK: AMP-activated protein kinase; FDX1: Ferredoxin 1; Hippo-YAP: Hippo-Yes-associated protein pathway; mTOR: Mechanistic target of rapamycin kinase; PI3K-IRF3-IFN-β: Interferon regulatory factor 3-interferon-β.

Accumulating evidence indicates that *FDX1* dysregulation influences tumor development through multiple mechanisms, including modulation of oncogenic signaling pathways, metabolic reprogramming, and alterations in tumor immunity [Figure 3].

### Signaling pathways

FDX1 is involved in several signaling pathways, including adenosine monophosphate-activated protein kinase (AMPK),^28^ mammalian target of rapamycin (mTOR),^28^ phosphatidylinositol 3′-kinase (PI3K)-AKT,^5^ IFN regulatory factor 3-interferon-β (IRF3-IFN-β),^29^ and Hippo-Yes-associated protein (Hippo-YAP).^30^

AMPK and mTOR are essential for maintaining metabolic homeostasis and enabling metabolic adaptability.^51^ Liu et al. reported that FDX1 enhances autophagy by activating AMPK and inhibiting mTOR, thereby facilitating ovarian cancer progression.^28^ Compound C, a selective AMPK inhibitor, effectively suppresses tumor growth in ovarian cancer models with *FDX1* overexpression.^28^ The PI3K-AKT pathway regulates tumor growth, drug resistance, invasion, and metastasis in various cancers.^52^ FDX1 knockdown activates this pathway by increasing reactive oxygen species (ROS) production, which enhances HCC growth and metastasis *in vivo*.^5^ By contrast, the PI3K-AKT pathway phosphorylates FDX1 to suppress its enzymatic activity in triple-negative breast cancer (TNBC).^39^ Combined treatment with AKT1 inhibitors and copper ionophores synergistically suppresses TNBC tumorigenesis *in vitro* and *in vivo*.^39^ These findings suggest a negative feedback loop between FDX1 and the PI3K-AKT pathway, highlighting a potential therapeutic target for cancer intervention. IFN-β, a cytokine of the type I interferon family, suppresses cancer progression by exerting direct antitumor effects and promoting immune responses.^53^ FDX1 has been demonstrated to activate the IFN-β signaling pathway by enhancing IRF3 phosphorylation, a key transcriptional activator of IFN-β, thereby inducing PANoptosis in diffuse large B-cell lymphoma cells following elesclomol treatment.^29^ These findings reveal an unrecognized functional role of FDX1 in elesclomol-induced cell death. The Hippo pathway functions as a tumor suppressor by regulating the activity of Yes-associated protein (YAP) and transcriptional coactivator with a PDZ-binding motif (TAZ) in a wide range of human cancers.^54^ FDX1 knockdown inhibits colorectal cancer progression by upregulating phosphorylated YAP levels and downregulating TAZ expression.^30^ The Hippo–YAP pathway inhibitor GA-017 effectively counteracts the inhibitory effects of FDX1 silencing on colorectal cancer progression.^30^ Overall, dysregulation of FDX1 influences the activity of multiple signaling pathways. Investigating the regulatory interactions between FDX1 and these pathways may enhance understanding of cancer progression mechanisms and support the development of potential anticancer strategies.

### Metabolic reprogramming

FDX1 is involved in multiple metabolic pathways, particularly those associated with lipid homeostasis and mitochondrial metabolism. Disruptions of lipid homeostasis have been linked to various pathological conditions, including cancer.^55^ Suppression or depletion of ATP-binding cassette transporter A1 (ABCA1) leads to cholesterol accumulation, which subsequently activates sterol regulatory element-binding proteins 1 and 2 (SREBP1/2), key transcription factors that regulate lipid homeostasis in tumor cells.^56^ FDX1 deficiency promotes abnormal lipid accumulation through modulation of the ABCA1–SREBP1/2 signaling pathway.^31^ Furthermore, the FDXR–p53 axis plays an important role in lipid homeostasis and tumor progression.^57^ Loss of FDXR and/or p53 induces lipid accumulation in tumor cells via the downregulation of ABCA1 and activation of SREBP1/2.^57^ As a functional component of the FDXR complex, FDX1 may contribute to lipid homeostasis within the FDXR–p53 axis by modulating the ABCA1–SREBP1/2 pathway.

Mitochondrial metabolism plays a pivotal role in tumor progression by supplying essential metabolites for macromolecular synthesis and generating ATP to meet the heightened metabolic demands of cancer cells.^58^ FDX1 serves as a central regulator of mitochondrial metabolism, primarily through its role in protein lipoylation. Through direct interaction with LIAS, FDX1 promotes the assembly of the LIAS-GCSH complex, initiating lipoylation of target proteins.^33^ This post-translational modification enhances the enzymatic activity of four critical mitochondrial metabolic complexes: pyruvate dehydrogenase, α-ketoglutarate dehydrogenase, branched-chain α-ketoacid dehydrogenase, and the glycine decarboxylase complex.^33^ These lipoylated complexes are integral to the TCA cycle, BCAA catabolism, and glycine metabolism. Cancer cells depend heavily on the TCA cycle for energy production and biosynthesis of cellular components.^59^ Therapeutic agents targeting this cycle have shown promising antitumor efficacy across multiple cancer types.^60^ BCAAs exhibit context-dependent roles in tumor progression, promoting proliferation by providing energy and biosynthetic intermediates in some cancers, while inducing cell cycle arrest or augmenting in immune-mediated tumor suppression others.^61^ Dysregulation of glycine cleavage metabolism also contributes to the progression of multiple cancers, including multiple myeloma^62^ and lung cancer,^63^ as demonstrated in both *in vitro* and *in vivo* studies. Given the critical role of these mitochondrial metabolic pathways, FDX1 may modulate cancer progression by regulating lipoylation-dependent metabolic processes.

*FDX1* expression is closely linked to glucose metabolism, fatty acid oxidation, oxidative phosphorylation, and amino acid metabolism across multiple cancer types.^64^ In addition, FDX1 regulates key metabolic enzymes, such as glucose-6-phosphate dehydrogenase^65^ and hypoxia-inducible factor 1α.^27^ However, the precise mechanisms through which FDX1 modulates these metabolic pathways and enzyme activities remain unclear. Collectively, these results indicate that FDX1 plays a crucial role in the metabolic reprogramming of tumor cells. Further research is necessary to clarify the specific regulatory mechanisms and their contributions to tumor metabolism.

### Tumor immunity

Despite significant advancements in immune checkpoint-based immunotherapy, only a subset of patients achieve durable clinical response, highlighting tumor immune evasion as a major obstacle to effective cancer treatment. Analysis of single-cell RNA sequencing data from 11 patients with glioma revealed that *FDX1* is expressed in most immune cells within the tumor microenvironment. Glioma patients with high *FDX1* expression also exhibit increased immune cell infiltration.^34^ In HCC tissues with elevated *FDX1* expression, infiltration of natural killer (NK) cells, macrophages, and B cells notably increases.^66^ In ccRCC, *FDX1* overexpression enhances antitumor immunity by promoting the secretion of interleukin-2 (IL-2) and interferon-γ (IFN-γ).^67^ Conversely, impaired FDX1 enzymatic activity correlates with reduced immune cell infiltration in ccRCC.^68^ IFN-β has been shown to promote immune cell recruitment, including CD8^+^ T-cell and NK cell infiltration.^69^ These findings suggest that FDX1 may facilitate immune cell infiltration by activating the IRF3-IFN-β signaling pathway. YAP, a key component of the Hippo pathway, is positively and negatively correlated with M2 macrophage and regulatory T-cell infiltration in melanoma, respectively.^70^ This suggests that FDX1 may modulate immune cell infiltration through regulation of the Hippo–YAP pathway. Collectively, these findings imply that FDX1 contributes to enhanced immune cell infiltration within the tumor microenvironment.

FDX1 also regulates the expression of immune checkpoint genes. In ccRCC, *FDX1* expression shows a positive correlation with several immune checkpoint genes, including programmed cell death ligand 1 (*PD-L1*), hepatitis A virus cellular receptor 2, and programmed cell death ligand 2. Conversely, *FDX1* exhibits a negative correlation with cytotoxic T-lymphocyte-associated protein 4 (CTLA-4), lymphocyte activation gene 3 (LAG-3), and programmed cell death 1 (*PD-1*).^71^ Glioma tissues with high *FDX1* expression demonstrate elevated levels of immune checkpoint markers, such as *PD-L1*, *CD40*, *CD80*, and *CD86*.^34^ silencing FDX1 reduces PD-L1 expression in melanoma cells.^72^ Several oncogenic signaling pathways, including PI3K–AKT–mTOR and NF-κB, regulate immune checkpoint gene expression.^73^^,^^74^ These findings suggest that FDX1 may modulate immune checkpoint gene expression by regulating these signaling pathways.

Metabolism in both cancer and immune cells critically shapes the tumor immune microenvironment. In cancer cells, metabolic reprogramming enables the production and release of immunomodulatory molecules, such as ATP, mitochondrial DNA, high mobility group box 1, and α-ketoglutarate, which modulate antitumor immune responses.^58^^,^^75^^,^^76^ Mitochondrial metabolism-derived ROS also activate key transcription factors that support optimal T cell function.^58^ Memory T cells primarily depend on oxidative phosphorylation (OXPHOS) to support their metabolic needs.^58^ *FDX1*, expressed in both tumor and immune cells within the tumor microenvironment, plays a central role in regulating metabolism through protein lipoylation.^34^ Therefore, *FDX1* may function as a metabolism-immune checkpoint gene through the dual regulation of metabolism in tumors and immune cells. Given its association with immune cell infiltration and immune checkpoint gene expression, *FDX1* expression may serve as a predictive biomarker for immunotherapy response. Modulating *FDX1* expression or activity could potentially enhance the efficacy of immune checkpoint inhibitors. Although correlations between *FDX1* expression and features of the tumor immune microenvironment have been observed, the underlying molecular mechanisms remain unclear.

### Molecular basis of ferredoxin 1 dual roles in cancer progression

Tumors undergo metabolic reprogramming that supports cancer progression.^77^ In mitochondrial metabolism-dependent cancers, FDX1 may promote tumor growth by enhancing mitochondrial enzyme activity through lipoylation. Meanwhile, FDX1 mediates cuproptosis and increases immune cell infiltration, potentially sensitizing tumors to cuproptosis inducers and immunotherapy. In cancers that rely on aerobic glycolysis, *FDX1* overexpression may suppress cancer progression by shifting cellular metabolism from aerobic glycolysis to OXPHOS. Additionally, cancer cells reprogram their metabolism to support their survival during tumor progression. In the early stages, aerobic glycolysis supports proliferation by providing energy and biosynthetic intermediates,^78^ whereas in advanced stages, OXPHOS contributes to drug resistance, radioresistance, and metastasis.^79^ Single-cell sequencing data reveal that *FDX1* expression increases from early to advanced stages in several cancer types,^44^^,^^45^ suggesting that FDX1 regulates this metabolic transition. Through the activation of OXPHOS to support energy supply for metastasis, FDX1 may promote tumor progression in advanced cancers while simultaneously increasing sensitivity to cuproptosis inducers. The mitochondrial metabolic profiles of cancer may therefore determine the functional role of FDX1 during cancer progression.

Autophagy suppresses tumorigenesis by limiting cancer cell survival; however, it can also promote resistance to chemotherapy and radiotherapy while facilitating tumor metastasis.^80^ Therefore, FDX1 may inhibit tumorigenesis yet promote tumor progression by inducing autophagy. Given that FDX1 serves as a key mediator of cuproptosis, copper levels within the tumor microenvironment may modulate its functional role in cancer progression. The liver, the primary organ for copper storage in the human body,^81^ enables FDX1 to function as a tumor suppressor in HCC by mediating cuproptosis. By contrast, the Hippo–YAP signaling pathway acts as a tumor suppressor in colorectal cancer,^82^ but promotes tumor progression in breast cancer,^83^ which may explain FDX1's tumor-promoting role in colorectal cancer and tumor-inhibiting function in breast cancer. These signaling pathways, therefore, contribute to the dual functions of FDX1 in cancer progression. Collectively, cancer type, metabolic characteristics, autophagy, copper levels in the tumor microenvironment, signaling pathways, and cuproptosis may contribute to the dual roles of FDX1 in cancer progression.

## Therapeutic potential of ferredoxin 1 (FDX1)

FDX1 plays a crucial role in the progression of multiple cancers and is a key mediator of cuproptosis. Targeting FDX1 is a promising therapeutic strategy for cancer treatment. Several recent approaches have been developed to directly or indirectly modulate FDX1 activity, demonstrating its therapeutic potential in *in vitro* and *in vivo* cancer models.

### Copper ionophores

The elesclomol-Cu^2+^ complex to directly bind to the α2/α3 helices and β5 strand of FDX1, with no detectable interaction with its paralog FDX2.^84^ This binding induces complete oxidation of FDX1 and promotes Cu^+^ generation, ultimately triggering cuproptosis. Substantial evidence indicates that elesclomol exerts anticancer effects through FDX1-mediated cuproptosis in various cancer types.^85^, ^86^, ^87^, ^88^ Disulfiram, another copper ionophore initially approved for alcohol aversion therapy, also exhibits promising anticancer activity by enhancing FDX1-mediated cuproptosis.^89^ The therapeutic efficacy of copper ionophores, either as monotherapy or in combination with other agents, has recently been evaluated in multiple cancer trials across different cancer types [Table 2]. However, most clinical trials have not achieved the desired clinical responses. Limited efficacy, safety concerns, and pharmacokinetic limitations contribute to these shortcomings.^90^ Both copper ionophores undergo rapid plasma clearance and exhibit short half-lives, making it difficult to maintain sustained therapeutic concentrations.^90^ Safety issues, such as hepatic copper accumulation, cardiotoxicity, and neurotoxicity, were major factors in the failure of the elesclomol clinical trial.^90^^,^^91^ Nanoparticle-based delivery systems for copper ionophores may improve selectivity and enhance accumulation within tumor tissues.^26^^,^^92^^,^^93^ Patients with melanoma and low lactate dehydrogenase (LDH) levels exhibit better responses to elesclomol than those with high LDH levels.^94^ Elevated tumor LDH is associated with hypoxia and reflects a glycolytic phenotype in cancer cells, supporting the therapeutic potential of copper ionophores in metabolic targeting strategies. Furthermore, combining copper ionophores with other anticancer agents that synergize with their mechanisms of action may increase their effectiveness. Several studies have found that copper ionophores exhibit increased potency when combined with glycolysis inhibitors,^95^ platinum compounds,^88^ or proteasome inhibitors.^85^

**Table 2:** Clinical trials of copper ionophores.

Cuproptosis-related drugs	Clinical trial phase	Study population	Main results	Clinical trials ID	References
Elesclomol in combination with paclitaxel	III	Metastatic melanoma patients	Effective in a subset of patients (*LDH* potentially a predictive marker for the combination regimen's suitability).	NCT00522834	94
Elesclomol sodium in combination with paclitaxel	II	Patients with metastatic melanoma, measurable disease, and one or fewer prior chemotherapy	Elesclomol sodium combined with paclitaxel significantly doubled median PFS, with acceptable toxicity and promising OS.	NCT00084214	123
Elesclomol sodium	I	Acute myeloid leukemia patients	Elesclomol showed good safety (max dose 400 mg/m^2^, no dose-limited toxicity) but no clinical responses in AML patients.	NCT01280786	124
Elesclomol sodium in combination with paclitaxel	II	Patients with platinum-resistant recurrent ovarian, tubal or peritoneal cancer	This combination was well tolerated but unworthy of further investigation due to response proportion.	NCT00888615	125
Elesclomol sodium in combination with paclitaxel	I	Adults with refractory solid tumors	The elesclomol/paclitaxel combination was well tolerated, with enhanced paclitaxel exposure (dose-dependently interacting with elesclomol) causing increased toxicity; responses in two pretreated patients support further evaluation.	NCT00088114	126
Elesclomol sodium in combination with taxane	II	Soft tissue sarcoma	Elesclomol enhanced taxane efficacy by induction of *HSP70*.	NCT00087997	127
Elesclomol sodium plus paclitaxel, carboplatin	I/II	Stage IIIB/IV non-small cell lung cancer	Unpublished	NCT00088088	N/A
Elesclomol sodium plus docetaxel and prednisone	I	Patients with metastatic prostate cancer	Unpublished	NCT00808418	N/A
Disulfiram/Cu	I	Patients with advanced solid tumors involving the liver	Disulfiram combined with copper gluconate was well-tolerated and safe in liver solid tumor patients. It led to temporary disease stabilization in some but no objective responses.	NCT00742911	128
Disulfiram/Cu	II	Metastatic breast cancer patients upon failure of conventional systemic and/or locoregional therapies	Unpublished	NCT03323346	N/A
Disulfiram/Cu in combination with cisplatin and vinorelbine	II	CTC_EMT positive refractory metastatic hormone receptor positive, HER2 negative breast cancer patients	Unpublished	NCT04265274	N/A
Disulfiram and cisplatin	II	Patients with multiple relapsed/refractory GCTs	Unpublished	NCT03950830	N/A
Disulfiram	NA	Recurrent prostate cancer patients	Unpublished	NCT01118741	N/A
Disulfiram/Cu	Ib	Pancreas cancer patients metastatic castrate-resistant prostate cancer patients	Unpublished	NCT02963051	N/A
Disulfiram in combination with cisplatin and vinorelbine	IIb	Patients with non-small cell lung cancer	The addition of disulfiram to a combination regimen of cisplatin and vinorelbine was well tolerated and appeared to prolong survival in patients with newly diagnosed non-small cell lung cancer.	NCT00312819	129
Disulfiram and cisplatin	NA	Patients with advanced gastric cancer	Unpublished	NCT05667415	N/A
Disulfiram in combination with chemotherapy	I	Patients with refractory solid tumors or metastatic pancreatic cancer	Unpublished	NCT02671890	N/A
Disulfiram and chelated zinc	II	Refractory disseminated malignant melanoma patients	Unpublished	NCT02101008	N/A
Disulfiram Plus arsenic Trioxide	I	Metastatic melanoma patients	Unpublished	NCT00571116	N/A
Disulfiram	I/II	Metastatic melanoma patients	Unpublished	NCT00256230	N/A
Disulfiram and metformin	I/II	Suspected glioblastoma or recurrent glioblastoma patients undergoing surgical resection	Unpublished	NCT03151772	N/A
Disulfiram/Cu with concurrent radiation therapy and TMZ	I/II	Patients with newly diagnosed glioblastoma	The combination regimen has limited clinical efficacy for most patients but shows promising efficacy in *BRAF*-mutant glioblastoma.	NCT02715609	130
Disulfiram/Cu in combination with TMZ	II	Recurrent TMZ-resistant glioblastoma patients	Adding disulfiram/Cu to TMZ for TMZ-resistant *IDH*-wild type glioblastoma is well tolerated, yet shows limited activity in an unselected population.	NCT03034135	131
Disulfiram/Cu	II	Newly diagnosed glioblastoma patients	Unpublished	NCT01777919	N/A
Disulfiram in combination with alkylating agents	I	Patients with glioblastoma	Unpublished	NCT01907165	N/A
Disulfiram in combination with temozolomide	II	Unmethylated glioblastoma patients	Unpublished	NCT03363659	N/A
Disulfiram/Cu in combination with alkylating chemotherapy	II/III	Patients with recurrent glioblastoma	For patients with recurrent glioblastoma, adding disulfiram and copper to chemotherapy increased toxic effects without improving survival.	NCT02678975	132
Disulfiram/Cu in combination with liposomal doxorubicin	I	Patients with treatment-refractory sarcomas	Unpublished	NCT05210374	N/A
Disulfiram/Cu	I	Patients with treatment-refractory multiple myeloma	Unpublished	NCT04521335	N/A
Disulfiram/Cu	II	Metastatic pancreatic cancer patients	Unpublished	NCT03714555	N/A

AML: Acute myeloid leukemia; *BRAF*: B-raf proto-oncogene, serine/threonine kinase; GCTs: Germ cell tumors; *HER2*: Erb-B2 receptor tyrosine kinase 2; *HSP70*: Heat shock protein 70; *IDH*: Isocitrate dehydrogenase; *LDH*: Lactate dehydrogenase; N/A: Not available; OS: Overall survival; PFS: Progression-free survival; TMZ: Temozolomide.

### Small-molecule-mediated upregulation or activation of ferredoxin 1 (FDX1)

Given the essential role of FDX1 in cuproptosis, increasing FDX1 levels or activity may provide an effective strategy for enhancing the sensitivity of cancer cells to cuproptosis inducers. The ferroptosis inducers sorafenib and erastin upregulate protein lipoylation by suppressing the mitochondrial matrix protease-mediated degradation of FDX1 and reducing intracellular glutathione synthesis by inhibiting cystine import, thereby inducing cuproptosis in liver cancer cells.^38^ Combining the elesclomol-Cu^2+^ complex with sorafenib or erastin synergistically suppresses liver cancer progression in xenograft mouse models by enhancing cuproptosis.^38^ [1-propyl-3,5-bis(2-bromobenzylidene)-4-piperidinone], a novel curcuminoid, effectively inhibits the proliferation, invasion, and migration of cervical cancer cells.^11^ Mechanistic studies have demonstrated that this curcuminoid upregulates *FDX1* expression by inhibiting the neurogenic locus notch homolog protein 1 (Notch1)–recombination signal-binding protein for the immunoglobulin kappa J region (RBP-J)–NRF2 signaling pathway, leading to cuproptosis in cervical cancer cells.^11^ In TNBC, AKT1 phosphorylates FDX1 protein and inhibits its activity, thereby inducing cuproptosis resistance.^39^ The combination of MK2206, a selective AKT1 inhibitor, with the elesclomol-Cu^2+^ complex synergistically suppresses TNBC tumorigenesis by enhancing cuproptosis *in vitro* and *in vivo*.^39^ Fluphenazine, a first-generation antipsychotic, induces cuproptosis by upregulating *FDX1* expression at both the mRNA and protein levels in breast cancer.^40^ Collectively, these findings suggest that enhancing *FDX1* expression or augmenting its protein activity is a promising therapeutic strategy.

### Combination immunotherapy

*FDX1* expression positively correlates with immune cell infiltration in several cancer types. Moreover, FDX1 regulates the expression of immune checkpoint genes,^34^^,^^35^^,^^66^^,^^72^ making it a potential predictor of the immunotherapy response in cancers, including ccRCC,^67^ HCC,^66^ glioma,^34^ and colon adenocarcinoma.^96^ Upregulation or activation of FDX1 may facilitate the conversion of “cold tumors” into “hot tumors,” which respond more effectively to immunotherapy. High *FDX1* expression has been associated with improved responses to PD-L1 blockade in melanoma.^72^ Drug sensitivity analyses revealed that *FDX1* expression is positively correlated with the response to PD-1 inhibitor therapy in patients with melanoma and kidney cancers.^97^ Combining an FDX1 activator with an immune checkpoint inhibitor may therefore offer a promising therapeutic approach. Recent studies demonstrate that copper ionophores combined with immune checkpoint inhibitors, such as anti-CD47 antibody, JQ-1, and anti-PD-1 (aPD1), yield significant therapeutic potential in multiple cancers.^93^^,^^98^^,^^99^ Given that FDX1 functions as a central regulator of both cuproptosis and the tumor immune microenvironment, it may act as an essential mediator of the synergistic effects observed between copper ionophores and immunotherapy checkpoint inhibitors.

### Other therapeutic strategies

Upregulation of *FDX1* expression has been observed in various forms of drug resistance and radioresistance across multiple cancer types,^15^ highlighting the therapeutic potential of cuproptosis in these malignancies. Under severe hypoxia, *FDX1* expression increases and promotes radioresistance in glioblastoma.^27^ Radiotherapy elevates mitochondrial copper levels by upregulating copper transporter 1 expression and reducing mitochondrial glutathione, thereby triggering cuproptosis in patient-derived xenografts.^100^ These findings indicate that radiotherapy may synergize with copper ionophores to overcome tumor radioresistance through FDX1-mediated cuproptosis. Moreover, the observed upregulation of FDX1 in tumors following radiotherapy supports the use of copper-containing nanocapsule-like polyoxometalates, which have demonstrated efficacy in overcoming acquired radioresistance, even at clinically relevant radiation doses, in preclinical tumor models.^26^ FDX1-mediated cuproptosis has been shown to overcome various forms of drug resistance in tumor cells and preclinical models, such as enzalutamide resistance in prostate cancer,^86^ cisplatin resistance in ovarian cancer,^25^ adrenomedullin-mediated sunitinib resistance in ccRCC,^13^ and lenvatinib resistance in HCC.^101^ Therefore, FDX1-mediated cuproptosis represents a promising therapeutic strategy for addressing both drug resistance and radioresistance in cancer.

The crosstalk among various regulated cell death pathways involves multiple key mediators,^102^ therefore, targeted modulation of these mediators represents a promising therapeutic strategy for cancer.^103^ In addition to cuproptosis, excessive copper induces other forms of cell death, including apoptosis, pyroptosis, and ferroptosis.^104^ Copper-induced ROS generation and proteotoxicity constitute the primary mechanisms underlying copper-mediated cytotoxicity. FDX1 reduces Cu^2+^ to Cu^+^, which subsequently enhances ROS generation via the Fenton reaction and leads to aggregation of lipoylated proteins. Therefore, FDX1 may play a pivotal role in mediating copper-induced cell death. A core-shell nanoparticle incorporating copper and erastin, a ferroptosis inducer, has demonstrated significant anticancer efficacy by synergistically activating both cuproptosis and ferroptosis in murine models of colon adenocarcinoma and TNBC.^105^ Similarly, a copper-based metal-organic framework (Cu-MOF) exhibits enhanced antitumor activity *in vitro* and *in vivo* through the co-activation of apoptosis and cuproptosis.^106^ These findings highlight the potential of combining cuproptosis with other regulated cell death pathways through engineered combination therapy nanoparticles.

Aggregation of lipoylated proteins is the primary mechanism underlying cuproptosis; therefore, upregulation of protein lipoylation-related genes, such as *LIAS*, dihydrolipoamide S-acetyltransferase (*DLAT*), *GCSH*, dihydrolipoamide s-succinyltransferase, and dihydrolipoamide branched-chain transacylase E2, may enhance cuproptosis in cancer. FDX1 regulates cellular protein lipoylation by directly binding to LIAS. Elevated *LIAS* expression in multiple cancers increases lipoylated protein levels, thereby enhancing susceptibility to cuproptosis. Deletion of *FDX1* or *LIAS* confers resistance to cuproptosis.^107^ *DLAT* is overexpressed and serves as a promising prognostic marker and therapeutic target in most cancers.^108^ FDX1-mediated accumulation of lipoylated DLAT aggregation is a key mechanism underlying cuproptosis in cancer cells. Given the critical roles of these genes, a gene signature comprising *FDX1* and other cuproptosis-related genes may serve as a biomarker for identifying cancers susceptible to cuproptosis.

## Conclusions and future perspectives

Overall, *FDX1* expression is dysregulated across various cancer types. Its expression is significantly associated with prognosis, disease progression, cuproptosis, and tumor immunity in multiple malignancies, highlighting its potential as a biomarker for cancer diagnosis, prognostic evaluation, and treatment decision-making. Several factors, including cancer type, metabolic characteristics, autophagy, copper levels within the tumor microenvironment, signaling pathways, and cuproptosis, may contribute to FDX1's dual roles in different cancers. Therapeutic strategies targeting FDX1 directly or indirectly have shown promising efficacy in multiple cancer models.

Enhanced expression or activity of FDX1 produces significant synergistic effects when combined with copper ionophores in various malignancies. Moreover, FDX1 influences cancer progression by modulating signaling pathways, metabolic reprogramming, and tumor immune responses. Dynamic modeling of signaling networks has elucidated key regulatory mechanisms and enabled the systematic identification of potential therapeutic targets.^109^ Understanding the upstream and downstream regulatory mechanisms of FDX1 may reveal novel strategies to enhance its expression or functional activity, thereby facilitating the development of innovative therapeutic approaches for cancer treatment. *FDX1* is expressed in tumor cells, immune cells, and fibroblasts; thus, single-cell RNA sequencing could elucidate its distinct functional roles within the tumor microenvironment.

Copper ionophores such as elesclomol and disulfiram demonstrate substantial anticancer activity in multiple preclinical models; however, their clinical efficacy in human trials remains limited. Virtual screening approaches based on the crystal structure of the FDX1-Cu^2+^ complex may aid in designing novel copper ionophores with improved translational potential for cuproptosis-targeted therapies. Patient-derived xenograft models have been widely used in preclinical research^38^^,^^100^ and provide clinically relevant platforms for advancing the development of cuproptosis inducers. Furthermore, combining copper ionophores with other anticancer agents may yield synergistic therapeutic effects. Collectively, further research is warranted to elucidate the mechanisms by which FDX1 modulates cancer progression and to clarify its functional implications.

## Limitations of the review

This review summarizes the regulatory mechanisms, functional roles, and therapeutic potential of FDX1 in cancer; however, several limitations must be acknowledged. First, the dysregulation of *FDX1* and its association with cancer prognosis are primarily based on data from the TCGA and GEO databases. The absence of protein-level validation compromises the reliability of the conclusions. Second, the clinical responses to copper ionophores in clinical trials remain limited, with most studies still relying on *in vitro* or animal models. The lack of efficacy, safety concerns, and pharmacokinetic limitations may contribute to the limited clinical response to copper ionophores. Third, the dual roles of FDX1 in different cancer types reduce the therapeutic value of targeting FDX1. Therefore, it is imperative to clarify the functional role of FDX1 in cancer and to develop both activators and inhibitors of FDX1. Addressing these challenges through immunohistochemical staining, improved delivery systems, combination therapy strategies, and the rational design of novel FDX1-targeting agents is crucial for advancing the clinical application of FDX1-targeted therapies.

## Authors contribution

Jiliang Xia, Fen He, and Hongyan Zhao wrote the manuscript; Ruixin Gao, Maoyou Lu, Siqi Wang, and Yu Chen created the figures and table; Jiliang Xia and Lijun Peng revised the manuscript. All authors have read and approved the final version of the manuscript.

## Ethics statement

None.

## Data availability statement

The supporting data for this paper comes from published materials that are properly referenced and are available online.

## Declaration of Generative AI and AI-assisted technologies in the writing process

The authors declare that generative artificial intelligence (AI) and AI assisted technologies were not used in the writing process or any other process during the preparation of this manuscript.

## Funding

This work was supported by the 10.13039/501100004735Natural Science Foundation of Hunan Province (No. 2023JJ10036), the PhD Scientific Research Start-up Fund of the 10.13039/501100009020University of South China (No. 200XQD075), the Scientific Research Project of Hunan Department of Education (No. 22B0452), the Innovation Platform Open Fund Project of Department of Education of Hunan Province (No. 20K110).

## Conflict of interest

The authors declare that they have no known competing financial interests or personal relationships that could have appeared to influence the work reported in this paper.
